# Analysis of potential dynamic concealed factors in the difficulty of lower third molar extraction

**DOI:** 10.4317/medoral.21211

**Published:** 2016-10-01

**Authors:** Pradeep Singh, Deepal-Haresh Ajmera, Shui-Sheng Xiao, Xiao-Zhu Yang, Xiong Liu, Bin Peng

**Affiliations:** 1 Chongqing key Laboratory of Oral Diseases and Biomedical Sciences, Chongqing, China; 2 MD. Department of Oral & Maxillofacial Surgery, College of Stomatology, Chongqing Medical University, Chongqing, China; 3 MD. Department of Orthodontics and Dentofacial Orthopedics, College of Stomatology, Chongqing Medical University, Chongqing, China; 4 MD, Associate Professor. Department of Oral Implantology, College of Stomatology, Chongqing Medical University, Chongqing, China; 5 MD, Professor. Department of Radiology and Imaging, College of Stomatology, Chongqing Medical University, Chongqing, China; 6 PhD, Professor. Department of Health Statistics, School of Public Health, Chongqing Medical University, Chongqing, China

## Abstract

**Background:**

The purpose of this study was to identify potential concealed variables associated with the difficulty of lower third molar (M3) extractions.

**Material and Methods:**

To address the research purpose, we implemented a prospective study and enrolled a sample of subjects presenting for M3 removal. Predictor variables were categorized into Group-I and Group-II, based on predetermined criteria. The primary outcome variable was the difficulty of extraction, measured as extraction time. Appropriate univariate and multivariate statistics were computed using ordinal logistic regression.

**Results:**

The sample comprised of 1235 subjects with a mean age of 29.49 +/- 8.92 years in Group-I and 26.20 +/- 11.55 years in Group-II subjects. The mean operating time per M3 extraction was 21.24 +/- 12.80 and 20.24 +/- 12.50 minutes for Group-I and Group-II subjects respectively. Three linear parameters including B-M2 height (distance between imaginary point B on the inferior border of mandibular body, and M2), lingual cortical thickness, bone density and one angular parameter including Rc-Cs angle (angle between ramus curvature and curve of spee), in addition to patient’s age, profile type, facial type, cant of occlusal plane, and decreased overbite, were found to be statistically associated (*P* < or = 0.05) with extraction difficulty under regression models.

**Conclusions:**

In conclusion, our study indicates that the difficulty of lower M3 extractions is possibly governed by morphological and biomechanical factors with substantial influence of myofunctional factors. Practical Implications: Preoperative evaluation of dynamic concealed factors may not only help in envisaging the difficulty and planning of surgical approach but might also help in better time management in clinical practice.

**Key words:**Third molar, impacted, extraction, mandibular, facial type.

## Introduction

The surgical removal of third molars has been, and continues to be, the most frequently performed operation by oral and maxillofacial surgeons, both in private practice and in hospital settings. Evidence from various epidemiological studies suggests that at least, one impacted M3 can be traced in 33% of the general population (1) and mandibular M3 is the most frequently impacted teeth among them. Numerous dental and skeletal factors including spatial relationships with the ascending ramus of the mandible and with the occlusal plane have been investigated that have a potential bearing on the predictability of complexity in mandibular M3 removal. An overview of the literature shows that significant differences exist in maxillary and mandibular M3 space availability and in M3 angulation between Class I and Class II malocclusions (2). Likewise, Richardson et al. (3) cited retrognathic skeletal base as an etiological factor in the development of impaction.

The surgical difficulty of lower M3 removal may vary from routine to complex, depending on a series of factors, with no direct relationship between surgical difficulty and level of experience, as reported by Komerik *et al.* (4). Previous studies have reported a variety of factors that have been associated with the difficulty of impacted mandibular M3 removal including depth, ramus relationship/space available, and width of the root (5). Besides, an association between increased surgical difficulty and prolonged recovery after M3 removal has been reported (6). The above-mentioned factors are considered to be the predictors of difficulty during disimpaction, and despite this fact, clinicians have experienced difficulties during the extraction procedure. Moreover, based on the above-mentioned predictors and preoperative assessment, impacted M3 that appears to be easy for removal in the panoramic radiograph, might offer varying difficulty levels intra-operatively, in the patients with different skeletal patterns. Now the question arises, whether there are some hidden dynamic factors that might influence the difficulty of impacted mandibular M3 removal? Therefore, we hypothesize that, apart from the pre-investigated predictive factors, certain dynamic concealed factors might also contribute to the difficulty of mandibular M3 extraction. With this intent, the present study was aimed to explore and evaluate the role of potential dynamic concealed factors and their association with intra-operative complexity of mandibular M3 removal. Although, a number of studies have investigated this association before, but, to the best of our knowledge this is the first study of its kind, comparing different profile types and facial types for exploration of potential dynamic concealed factors in the difficulty of lower M3 removal.

## Material and Methods

- Selection of Subjects

The designed study included a total of 1235 unrelated Chinese subjects of Asian descent and Han ethnicity, with impacted mandibular M3, recruited from the Department of Oral & Maxillofacial Surgery, over a period of 7 months between November 2014 and June 2015. The study protocol was approved by the Ethics Committee of the University and informed written consents were obtained from all the eligible subjects, in compliance with the Principles of the Helsinki Declaration, before commencing this study. All patients fulfilled the following inclusion criteria: (a) aged 18 to 45 years; (b) presented with an impacted mandibular M3 (Left or Right side) and underwent a surgical extraction; (c) absence of any other craniofacial anomaly or systemic disease. None of the subjects had a history or current manifestation of systemic conditions that could modify the exodontia status, including diabetes, cancer, cardiovascular diseases, respiratory diseases, or transmissible infectious diseases (HIV or Hepatitis). Exclusion criteria also included patients presenting with pregnancy, immune-compromised patients and patient with Distoangular, Class-III, and Position-C impacted M3 positions (7,8).

**Figure 1:**
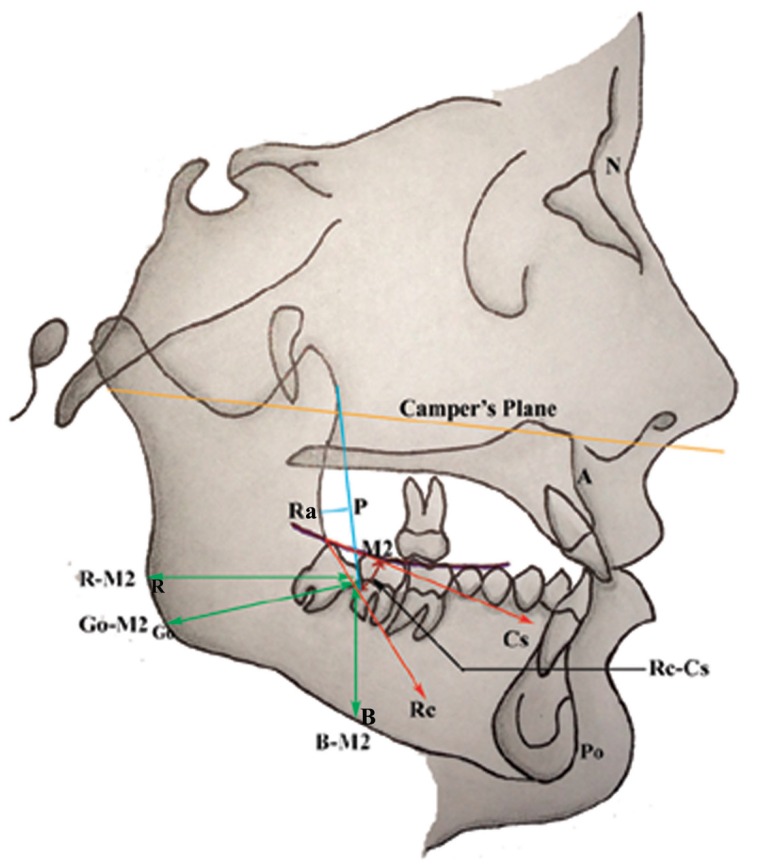
Skeletal landmarks and measurements (linear and angular) utilized on lateral cephalogram. N, nasion; A, subspinale; Po, pogonion; Go, gonion; Ra, deepest point on ramus curvature; P, perpendicular drawn from Ra; Ra-P, distance between Ra and P; Rc, tangent drawn along ramus curvature; Cs, tangent drawn along Curve of Spee; Rc-Cs, angle between ramus curvature and curve of spee; M2, point on the distal surface of second molar where ramus curvature meets with the distal surface; R-M2, distance between posterior border of ramus and M2; Go-M2, distance between Go and M2; B-M2, distance between inferior border of mandibular body and M2, Camper’s plane used in the study.

After pre-operative assessment and careful examination, subjects were divided into two groups (Group-I and Group-II), designed for the study, based on their lateral profile and frontal view. On the basis of reference points namely; [1] Nasion (N); [2] Subspinale (A); [3] Pogonion (Po) (Fig. 1) the subjects in the Group-I (n=631) were divided into (a) Convex profile; (b) Concave profile; and (c) Straight profile profile types.

The Cases group comprised 425 patients with a mean age of 28.78 ± 8.68 years while the Controlled population was represented by 206 patients (mean age 30.96 ± 9.25 years) in Group-I ( Table 1).

**Table 1:**
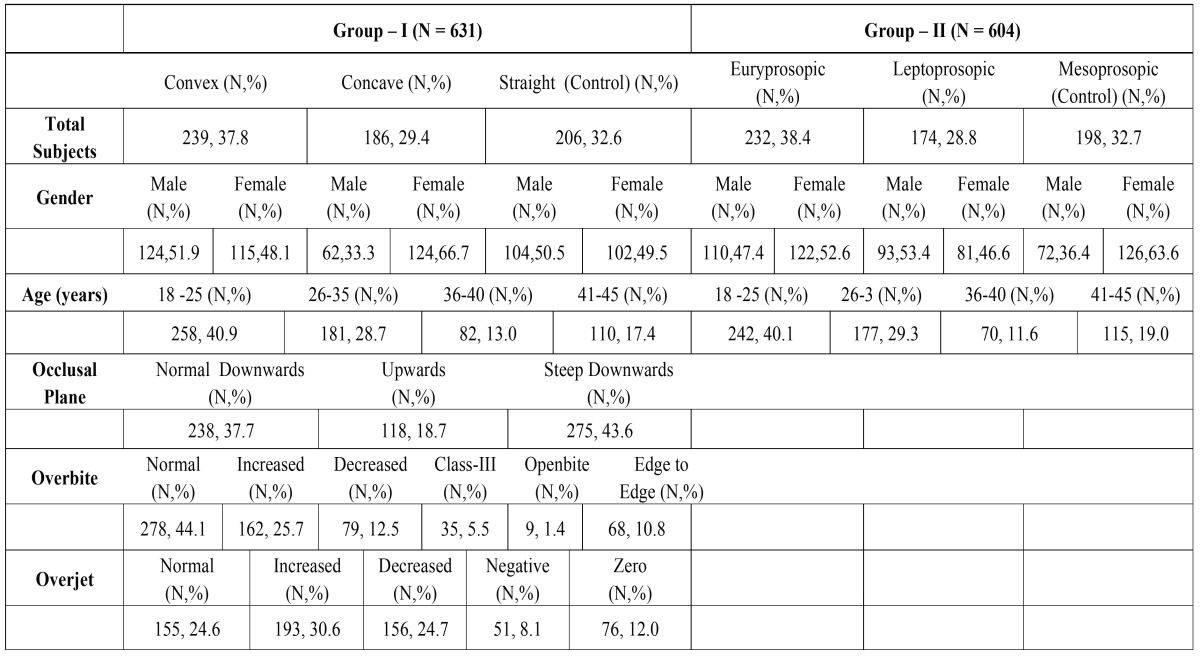
Demographical and Clinical characteristics patients by Profile type and Facial type.

Likewise, the sample for the second part of the study (Group –II; n=604) consisted of subjects with different facial types, which were divided into (a) Euryprosopic; (b) Leptoproposic; and (c) Mesoprosopic, according to anthropometric facial indices (9).

The Cases population in Group-II was represented by 406 patients with a mean age of 30.13 ± 9.10 years whereas the Controlled group included 198 patients (mean age 29.15 ± 8.67 years). Straight profile and Mesoprosopic subjects were considered to be Control group in Group-I and Group-II respectively. The baseline characteristics of the study population are listed in  Table 1.

- Baseline Investigated Parameters

Group-I - The maxillofacial morphology of subjects was assessed by measurements recorded indirectly from standardized Lateral cephalometric (LC) radiographs. LC radiographs were obtained for each participant in centric occlusion with the lips in repose and the Frankfort plane, horizontal, according to the natural head position, using a Kavo Pan eXam® Plus (Palo DEx Group Oy, Finland) cephalostat at 57 - 90 kVp, 16 mA and 10 seconds of scanning time. N-A-Po angle determined by LC radiographs was used to allocate subjects into their respective groups. LC radiographs were traced manually in a darkened room on acetate tracing paper using a 0.3 mm HB mechanical pencil. Subsequently, four linear (Go-M2, R-M2, B-M2, Ra-P) and one angular (Rc-Cs) measurements were recorded. Measurements were performed manually using a ruler to the nearest 0.1 mm. The radiographic reference points closely followed those defined by Down *et al.* (10). A summary of the reference points and cephalometric measurements used in this study is shown in (Fig. 1). Owing to the fact that different facial profiles are associated with varying degrees of occlusal plane, overjet, and overbite, these parameters were examined clinically for further subgroup analysis. Although, SN (Sella-Nasion) Plane is noted in the dental literature as a standard objective leveling reference. However, for the purpose of investigating the position of the occlusal plane in the facial context, Camper’s plane was used in the present study because of its ease of identification and was defined as passing through right and left Tragus and Subnasale landmarks (Fig. 1). Also, the occlusal plane was identified by three landmarks: inter-incisal point of the upper central incisors and mesiobuccal cusps of the first upper right and left molars. Moreover, ‘normal’ occlusal plane for this study, was believed to be parallel to Camper’s Plane, sloping downward and forward. Furthermore, 2-3mm for overjet and 1-2mm for overbite was considered to be ‘normal’ for this study.

**Figure 2:**
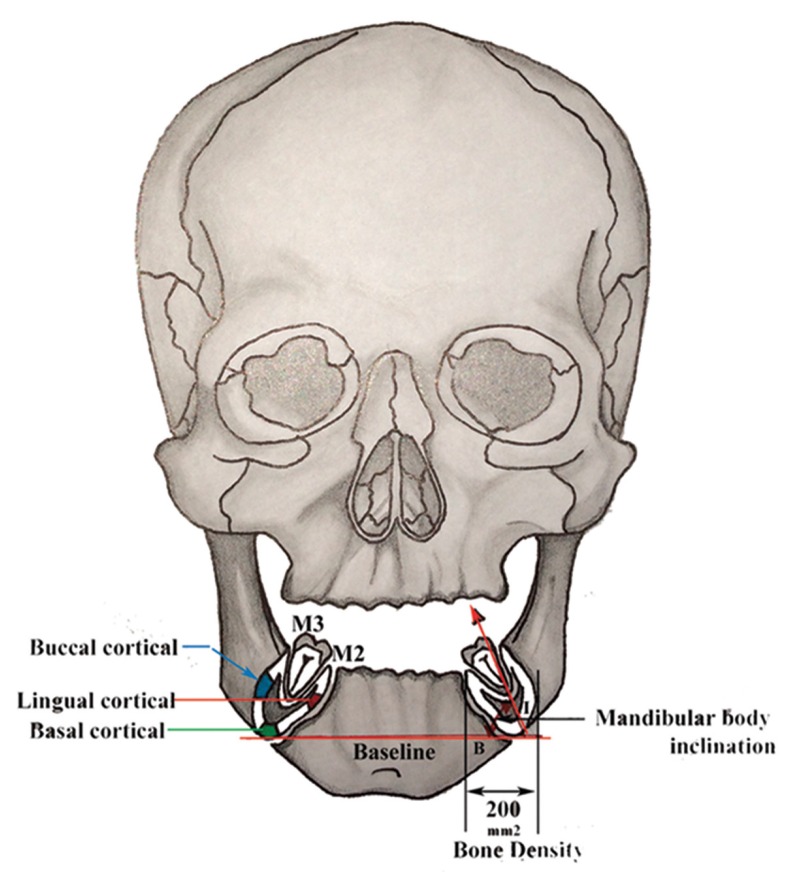
Reference points and parameters (linear and angular) analysed in the study. M3, third molar; M2, distal surface of second molar; mandibular cortical bone thickness (buccal, lingual, and basal); B-I, angle between mandibular baseline and bucco-lingual inclination of mandibular body; 200mm2, bony area between distal surface of second molar and third molar where bone density was analyzed.

Group-II- For each subject CBCT (KaVo 3D eXam, Germany) coronal view scans of the mandibular body were obtained for the analysis. These CT scans (1-mm slice thickness; 8.5 seconds scanning time; 120 Kvp; 3-8mA) were made in high-precision mode. The guidelines of the second molar (M2) sections were defined through the distal surface of the second molar in the lateral view. For the purpose of subject allocation with respect to predesigned groups, following five parameters (four linear and one angular) were measured in the coronal CBCT section for each patient: Cortical bone thickness (Buccal, Lingual, Basal); Bone density; and Bucco-lingual inclination of mandibular body. The inclination of mandibular body was assessed based on the angle formed by mandibular baseline and the line connecting the lowest point of the mandible and the midpoint of the buccolingual alveolar process peaks (Fig. 2). Landmarks were traced from each film following the same approach as in Group-I. Distances and angles were measured manually using reference points and variables as defined in fig. 2. Correspondingly, KaVo 3D eXamVision software was used to measure Cortical bone thickness; and Bone density. Besides, it is worth mentioning that Bone Density was measured in coronal CBCT view for an area of 200 mm2 distal to M2.

Surgical extractions were performed by seven independent oral surgeons (6 males and 1 female), with equivalent experience and skills, and the time utilized during disimpaction procedure was recorded. Furthermore procedural experience of the clinician, in terms of any kind of soft tissue interference (caused by tongue, or, buccal or retromolar soft tissues) encountered during the procedure, was also recorded.

- Statistical Analysis

Data analysis was carried out using the Statistical Package for Social Sciences (version 20.0; SPSS Inc., Chicago, Illinois, USA). Descriptive statistics were obtained for the 5 aforementioned variables of Group-I and Group-II respectively that included frequency, Mean and 95% CI for Mean ( Table 2). Intergroup differences were estimated by one-way analysis of variance (ANOVA) followed by the Tukey HSD test. In addition, a subgroup analysis for Group-I subjects was performed for variables like cant of Occlusal plane; Overjet; and Overbite. We also used proportional-odds ordinal logistic regression models that allowed us to compare multiple outcome categories in order to assess the independent effect of various parameters on the difficulty of M3 extraction. For the purpose of ordinal regression analysis, we divided the outcome variable into following methodological components: [1] Easy: < 30 minutes + No soft tissue interference [2] Difficult: > 30 minutes + No soft tissue interference [3] Very Difficult: > 30 minutes + Soft tissue interference. The strength of the association between various parameters and the difficulty level in extraction was evaluated by their ‘p’ values and odds ratios (ORs) under 2 regression models. Initially, a ‘pooled model’ was used which consisted of all the factors included in the study and their association with the difficulty in extraction. Subsequently, an ‘adjusted model’ was used to identify independent determinants of the difficulty in extraction, which included only those variables for which the ‘p’value in the univariate analysis was below 0.10. A p-value of less than 0.05 was considered statistically significant for all the tests. Furthermore, statistical analysis also included Pearson’s goodness of fit test.

**Table 2:**
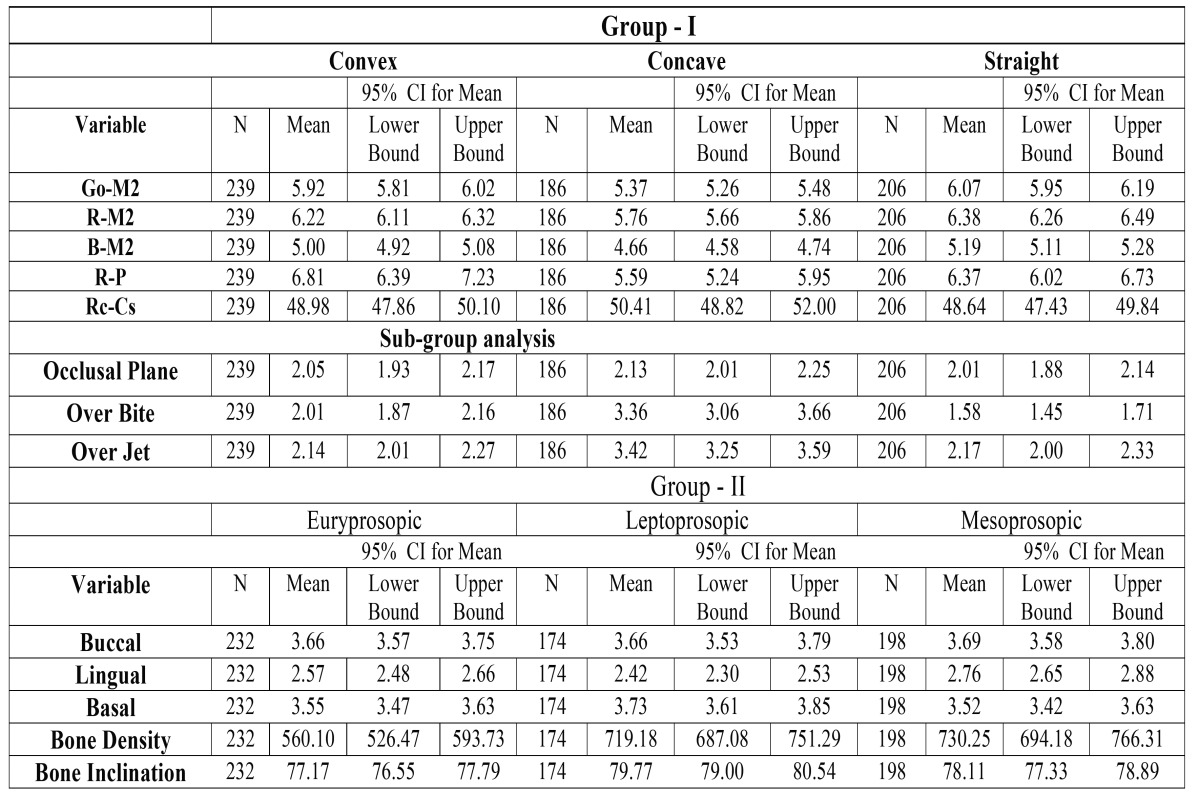
Descriptive statistics and statistical comparison of measurements in Group-I and Group-II subjects.

## Results

Demographical and clinical characteristics of the study subjects are illustrated in  Table 1. Further descriptive statistics for each measurement in Group-I and Group-II are summarized in  Table 2.

- Group-I

 Table 3 summarizes the results of ANOVA and post hoc test. Objective measurements of different parameters for Group-I subjects showed statistically significant differences between different profile types for Go-M2 ( F = 38.74, *p* = 0.000), R-M2 (F = 31.68, *p* = 0.000), B-M2 (F = 39.07, *p* = 0.000), and Ra-P (F = 9.67, *p* = 0.000). A Tukey post-hoc test revealed that Go-M2, R-M2 and Ra-P lengths are statistically significant in Convex ( *p* = 0.000, *p* = 0.000, and *p* = 0.000 respectively) and Straight (*p* = 0.000, *p* = 0.000, and *p* = 0.019 respectively) profile group compared with Concave profile. Besides, when B-M2 length was analyzed using post hoc test, significant differences were observed between all the three profile groups (*p* = 0.000, *p* = 0.000, *p* = 0.002).

**Table 3:**
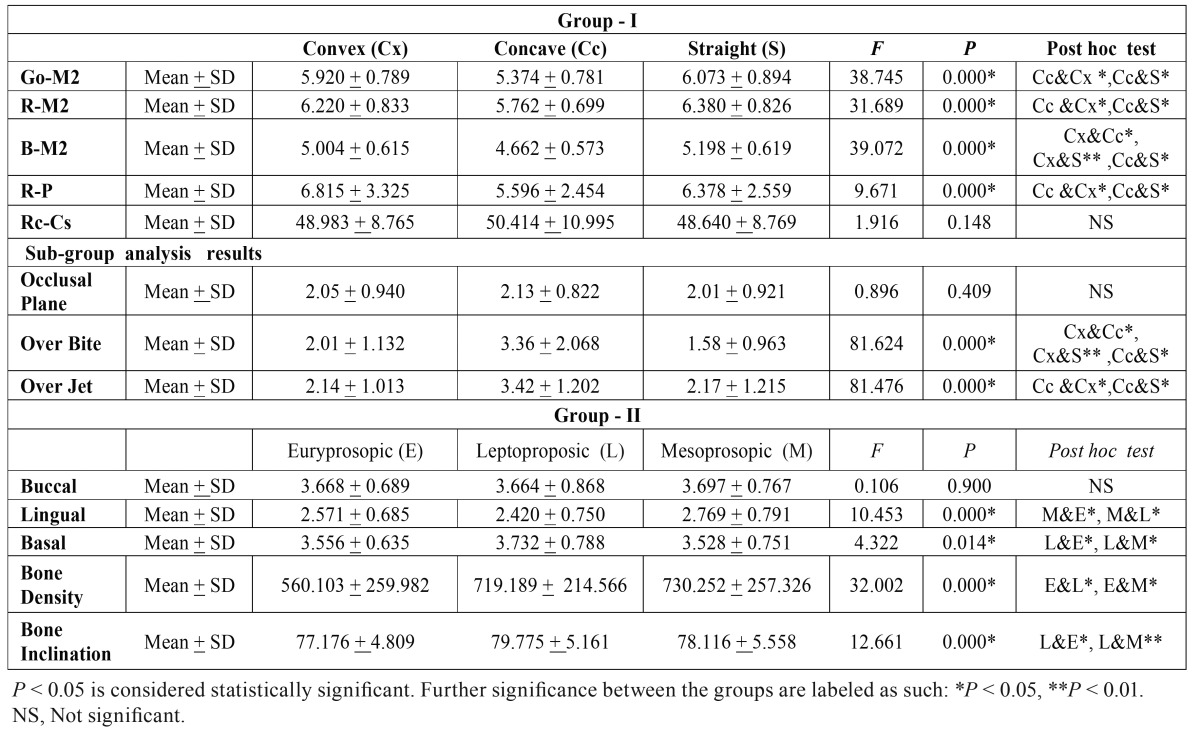
Overall results of Group-I and Group-II subjects for comparing various parameters in the Facial Profile and Facial Type view respectively.

- Sub-group analysis

Considering the influence of parameters like cant of occlusal plane, overjet and overbite on extraction difficulty, we also performed a subgroup analysis for Group-I subjects and endeavored to evaluate all the possible conditions for these parameters, which revealed statistically significant differences between the profile types when analyzed for overbite (F = 81.62, *p* = 0.000), and overjet (F = 81.47, *p* = 0.000). As shown in  Table 3, subjects with Convex and Straight profile showed significant differences for overjet (*p* = 0.000,and *p* = 0.000) when compared with Concave profile type. Also, post hoc test results for overbite revealed significant differences between all the three profile groups (*p* = 0.000, *p* = 0.000, *p* = 0.005).

- Ordinal logistic regression analysis

A further multiple ordinal logistic regression analysis was performed to evaluate the association of various parameters and difficulty of extraction. Odds ratios (OR) with their 95% confidence intervals (CI), Wald statistic and *p* values for the predictor variables are demonstrated in  Table 4. Multivariate analysis revealed the association of B-M2 (OR = 0.47, *p* = 0.010) with difficulty in disimpaction. Moreover, cant of occlusal plane (normal downwards, OR = 41.67, *p* = 0.000; and anteriorly upwards, OR = 534.85, *p* = 0.000), and decreased overbite (OR = 0.13, *p* = 0.034) also showed association with difficulty in extraction. Interestingly, results of our pooled model clearly show that it is difficult to extract lower M3 in patients with convex (OR = 0.31, *p* = 0.002) or concave profile (OR = 0.18, *p* = 0.000) as compared to straight profile. Furthermore, after exclusion of confounding factors such as gender, and also the covariates (Go-M2, R-M2, and B-M2) we found that Rc-Cs (OR = 1.02, *p* = 0.042) is also associated with the difficulty in removal of lower M3. Besides, as observed in pooled model, parameters like cant of occlusal plane (normal downwards, OR = 34.84, *p* = 0.000; and anteriorly upwards, OR = 426.66, *p* = 0.000) and decreased overbite (OR = 0.12, *p* = 0.031) were found to be associated with difficulty in extraction in the adjusted model too. Likewise, convex and concave profile type (OR = 0.33, *p* = 0.003; and OR = 0.20, *p* = 0.000 respectively) also showed significant association in the adjusted model. However, in contrast to the pooled model, B-M2, failed to show statistically significant results in the adjusted model.

**Table 4:**
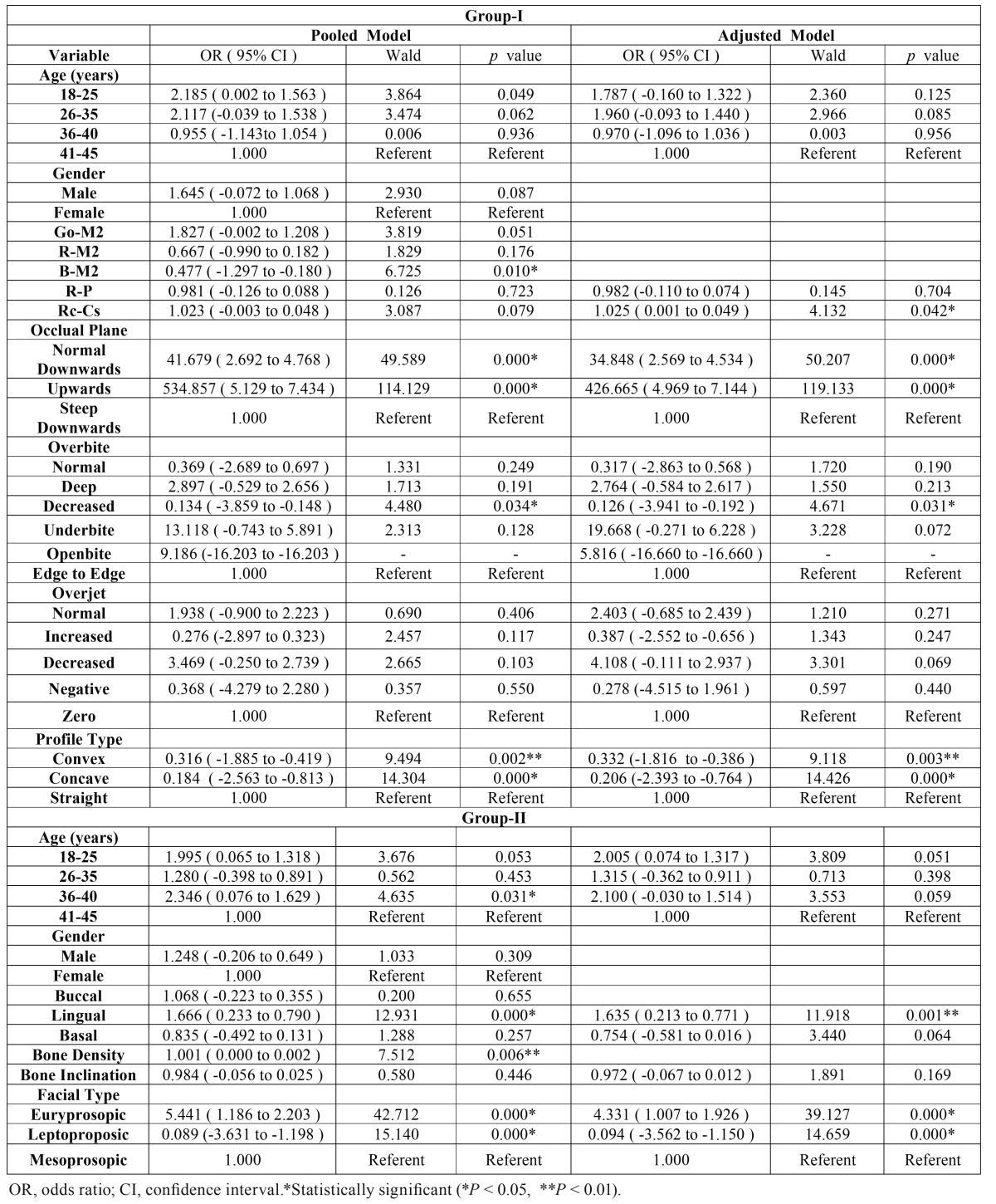
Multivariate ordinal logistic regression analysis for association between potential concealed factors and difficulty of lower M3 extraction.

- Group-II

The results of ANOVA and post hoc test are listed in  Table 3. A similar methodology was used for statistical analysis of Group-II subjects. The one-way analysis of variance showed statistically significant differences between different Facial types for parameters including cortical bone thickness (Lingual, F =10.45, *p* = 0.000; Basal, F = 4.32, *p* = 0.014), Bone Density (F = 32.00, *p* = 0.000), and Bucco-lingual inclination of mandibular body (F = 12.66, *p* = 0.000). Besides, Tukey HSD post hoc test revealed that Lingual cortical thickness was statistically significant in Euryprosopic (*p* = 0.016) and Leptoproposic (*p* = 0.000) facial types when compared with Mesoprosopic type. Likewise, Basal cortical thickness and Bucco-lingual bone inclination were found to be significant in Euryprosopic (*p* = 0.041, and *p* = 0.000 respectively) and Mesoprosopic (*p* = 0.018, and *p* = 0.006 respectively) facial types when compared with Leptoproposic type. Moreover, with respect to Bone density, statistically, significant differences were observed in Leptoproposic (*p* = 0.000) and Mesoprosopic (*p* = 0.000) facial types when analyzed against Euryprosopic type.

- Ordinal logistic regression analysis

To assess the potential association of specific study characteristics with difficulty in lower M3 removal, a multiple ordinal logistic regression analysis was performed, similar to Group-I. The results of multivariate analysis for the predictor variables investigated in the study are shown in table 4. In the pooled analysis, we found that age (36 to 40, OR = 2.34, *p* = 0.031) has a significant impact on the extraction difficulty. Besides, Lingual cortex, and Bone density (OR = 1.66, *p* = 0.000; and OR = 1.00, *p* = 0.006 respectively) were also found to be associated with lower M3 removal difficulty. Intriguingly, different facial types (Euryprosopic, OR = 5.44, *p* = 0.000; and Leptoproposic, OR = 0.08, *p* = 0.000) also showed significant association with extraction difficulty. Furthermore, the results of adjusted model were consistent with the pooled model, indicating the possible association of Lingual cortical thickness (OR = 1.63, *p* = 0.001), and facial types (Euryprosopic, OR = 4.33, *p* = 0.000; and Leptoproposic, OR = 0.09, *p* = 0.000) with lower M3 extraction difficulty. Conversely, age group (36 to 40) failed to show significant association in adjusted model, as against the pooled model.

## Discussion

Down *et al.* (10) in their study proposed that as the profile pattern deviates from straight towards convex or concave, the prognosis of satisfactory treatment result decreases. From the perspective of various skeletal patterns investigated in this study, the results have been promising and are in accordance with Down *et al.* (10) findings. Intriguingly, we found that different profile types and facial types are associated with lower M3 extraction difficulty under both the regression models. The underlying mechanism responsible for this association is unknown. However, the influence of masticatory musculature on the craniofacial growth might elucidate this association (11). In this regard, Satiroglu *et al.* (12), Benington *et al.* (13), and found that individuals with thick masseter had a vertically shorter facial pattern and individuals with thin masseter have a long face. Therefore, the effect of the muscle function on the form, growth, and structure of the mandible (14) can be substantiated to the fact that, thick and strong mandibular elevator musculature, as seen in Euryprosopic individuals, cause an increased mechanical loading of the jaws, followed by introduction of sutural growth and bone apposition, subsequently resulting in increased transverse growth of the jaws and bone bases. Besides, any alteration of the masticatory functional and mechanical demands might lead to region specific Bone mineral density (BMD) and bite force level changes in the mandible during growth (15), thereby affecting facial bone physiology and morphology (16). This explanation can be well attributed to the presence of significant differences in B-M2 lengths observed in our study, among subjects with different skeletal patterns. Such functional adaptive response by the mandible to mechanical stress resulting from mastication occurs not only in the muscle insertion area but also in mandibular alveolar bone in the molar region (17). Therefore, it can be suggested that myofunctional alterations due to variable skeletal patterns, would affect mandibular overall resistance to mechanical load, with the consequences on duration of extraction. Another factor that can be attributed to the above-mentioned association is the presence of significant differences between the interocclusal space of skeletal class II and class III patients. According to the pilot study performed by Loh *et al.* (18), interocclusal space may be less for class-III patient as compared to class-I patient, which is in agreement with the findings of our study.

An overview of literature suggests that masticatory function might play a substantial role in bone density, which had an imperative impact on extraction difficulty in the present study. A logical explanation for this association would be, since mandibular bone has a distinct feature associated with a noticeably great resistance to quasi-static loads imposed by the masticatory function, the higher the intensity of the applied load, the greater must be the cross-sectional area of bone tissue to resist the deformation. Moreover, cortical bone deposition on the periosteal surface, stimulated by mechanical load, increases bone resistance to reduce the peak strain to a level that does not compromise tissue integrity (19,20). Consequently, it can implied that increased region specific bone mineral density and bone resistance, might offer difficulty in extraction. Furthermore, studies have shown that masticatory musculature might also contribute in Median mandibular flexure (MMF) (21), caused by the functional contraction of the lateral pterygoid muscle and characterized by decrease in the arch width during jaw opening. This kind of mandibular deformation is maximum in Euryprosopic type patients, as concluded in Prasad *et al.* (21) study. Hence, it can be inferred that in Euryprosopic patients during jaw opening, arch width is decreased to a considerable extent as compared to other facial types. This is turn might lead to additional tongue interference, resulting in increased extraction time.

As indicated from the previous findings, significant differences exist in inclination of occlusal plane between skeletal class I, class II and class III (22), which led us to the assumption that occlusal plane might contribute to lower third molar removal difficulty. In this regard, Ogawa *et al.* (23) reported gliding or grinding type masticatory pattern predominately in cases with flat or posteriorly inclined occlusal planes. Therefore, we suggest that such kind of masticatory pattern might lead to increase in localized bone density, thereby adding to the extraction difficulty. Further, results of our analysis are in accordance with our assumption, which revealed a significant association between cant of occlusal plane and difficulty of lower M3 removal under both the regression models.

Pertaining to the influence of Rc-Cs on extraction difficulty, we observed statistically significant correlation, which can be elucidated by the fact that, the forward inclination of the superficial masseter muscle is responsible for forward tilting of molar teeth in the sagittal plane, that conforms to the posterior end of the curve of Spee. This tilt of the curve of Spee increases the crush/shear ratio of the force produced on food between the posterior molars (24). In addition, the concave border of anterior ramus favors the development of compression and tension loads during mastication. These natural bone curvatures tend to amplify the functional stimulus produced by mechanical loads, thereby restricting tissue deformation during mechanical load, as a result, bone tissue becomes more adapted and resistant to a normal pattern of strain distribution (25). In the present study also, significant differences were observed in ramus curvatures (Ra-P) among Group-I subjects which are in consensus with the previous findings. Hence, considering that the mandible receives intense mechanical load during extraction procedure, it may be implied that, with the change in Rc-Cs value, bone tissue resistance to extraction, also changes accordingly.

Cortical bone thickness seems to be influenced by the masticatory function and skeletal patterns as suggested by Masumoto *et al.* in their study (26). Moreover, a previous study by Tsunori *et al.* (27) suggested that thicker the buccal cortical bone, larger the facial height. Besides, Flanagan *et al.* (28) believed that mandibular lingual cortex is thicker as compared to facial cortex and there is some consistency to the lingual cortical predominance to provide functional osseous strength and stability. Apparently the results of our study are in agreement with the previous findings. The exact mechanism behind this association is not known. However, we can relate this association with Flanagan’s study and also to the fact that since most of the clinicians prefer a lingually directed path of removal for lower M3, therefore as the thickness of lingual cortical bone increases, difficulty in extraction increases proportionally.

Interestingly, results of subgroup analysis showed a significant association between difficulty level and decreased overbite condition under both the regression models. The exact mechanism behind such association is unclear. Further, a noteworthy finding of this analysis was the association of age group (36 to 40) with extraction difficulty in Group–II subjects. In this regard, Renton *et al.* (29) have linked increased bone density with the positive relationship between increased age and surgical difficulty, which was in accordance with the findings of our study to some extent, as we did not find any progressive increase in the surgical difficulty with age, thus indicating towards the possible influence of some other factors also. In the end, we can advocate that patients in age group of 36 to 40, with decreased overbite condition, have a clinical implication in the extraction difficulty.

The strengths of this study include a comprehensive examination of the subjects, applying explicit criteria to potentially eligible participants, and employing extracted data for the assessment of potential hidden factors. However, some limitations should be considered for our study. Firstly, we excluded Distoangular, Class-III, and Position-C impacted M3 from our study assuming them to be difficult enough to be extracted under local anesthesia, which could bring potential bias for our study. Secondly, we included only one ethnic group (Asian) in the present paper, thus, limiting the generalizability of our results to other populations, since different ethnic groups have different facial proportions. Further studies on this topic with larger sample sizes and in different ethnicities are expected to be conducted to strengthen our results.

## Conclusions

Within the limitations of this study, following conclusions can be drawn from this study,

1. Euryprosopic or Leptoprosopic facial type patients in age range of 36 to 40 years might offer substantial difficulty to lower M3 extraction.

2. Patient’s profile (Convex or Concave) might contribute to lower M3 extraction difficulty.

3. Extraction of lower M3 in patients with Normal downward and anteriorly upward directed cant of occlusal plane might be cumbersome.

4. Decreased overbite, B-M2 length, Rc-Cs angle, Lingual cortical bone thickness and Bone density have a noteworthy contribution in M3 extraction difficulty.

Thus, the epigenetic influence of masticatory muscles, as force-generating elements, on craniofacial growth, might be a valid explanation for the presence of positive findings in our study. Moreover, we presume that these skeletal factors can be used preoperatively to envisage the difficulty associated with the removal of impacted mandibular third molars. These findings may not only help in the prediction, and planning of surgical approach but may also help in better evaluation of treatment outcomes. Further avenues of research are needed to clarify the role of skeletal factors and for more substantiated results in other populations.
